# Connectivity-Based Parcellation of the Amygdala Predicts Social Skills in Adolescents with Autism Spectrum Disorder

**DOI:** 10.1007/s10803-017-3370-3

**Published:** 2017-11-08

**Authors:** Annika Rausch, Wei Zhang, Christian F. Beckmann, Jan K. Buitelaar, Wouter B. Groen, Koen V. Haak

**Affiliations:** 1 Department of Cognitive Neuroscience, Donders Institute for Brain, Cognition and Behaviour, Radboud University Medical Center Nijmegen, P. O. Box 9101, 6500 HB Nijmegen, The Netherlands; 2 Donders Institute for Brain, Cognition and Behaviour, Centre for Cognitive Neuroimaging, Radboud University, 6525 EN Nijmegen, The Netherlands; 3 Behavioural Science Institute, Radboud University, 6525 HR Nijmegen, The Netherlands; 4 Centre for Functional MRI of the Brain (FMRIB), University of Oxford, OX3 9DU Oxford, UK; 5 Karakter Child and Adolescent Psychiatry University Center, 6500 HB Nijmegen, The Netherlands

**Keywords:** Amygdala, Autism spectrum disorder, Functional connectivity, Parcellation, Prefrontal

## Abstract

Amygdala dysfunction plays a role in the social impairments in autism spectrum disorders (ASD), but it is unclear which of its subregions are abnormal in ASD. This study compared the volume and functional connectivity (FC) strength of three FC-defined amygdala subregions between ASD and controls, and assessed their relation to social skills in ASD. A subregion associated with the social perception network was enlarged in ASD (F_1_ = 7.842, *p* = .008) and its volume correlated significantly with symptom severity (social skills: *r* = .548, *p* = .009). Posthoc analysis revealed that the enlargement was driven by the vmPFC amygdala network. These findings refine our understanding of abnormal amygdala connectivity in ASD and may inform future strategies for therapeutic interventions targeting the amygdalofrontal pathway.

## Introduction

Autism spectrum disorders (ASD) are a group of neuro developmental disorders characterized by severe impairments of reciprocal social interaction, verbal and nonverbal communication, repetitive and stereotyped behaviors and abnormal sensory processes (American Psychiatric Association 2013; American Psychiatric Association 2000). The amygdala is thought to play a crucial role in the function of the ‘social brain’ in terms of being involved in social cognition, emotion recognition, socio-communicative perception and the regulation of emotional responses (Phelps and LeDoux 2005). The amygdala theory (AT) for ASD therefore hypothesizes that amygdala dysfunction underlies the social deficits seen in ASD (Baron-Cohen et al. 2000). In line with the AT’s predictions, the amygdala as a whole shows aberrant structural growth trajectories, exhibits abnormal functional connectivity (FC), and is involved in impaired emotion recognition and over-reactivity to aversive stimuli (Bellani et al. 2013; Green et al. 2013; Harms et al. 2010). The amygdala is, however, a composite structure, and its three major nuclei—the laterobasal, superficial and centromedial nuclei—have many connections with a wide variety of cortical areas. Yet, little is known about which amygdala pathways within the ‘social brain network’ (SBN) are compromised in ASD.

In earlier work, we used three anatomically defined amygdala subdivisions as seeds for a FC strength analysis and demonstrated that alterations within the amygdala network in ASD can be traced down to specific amygdala subdivisions. This approach, however, reveals functional connections that are associated with each amygdala subdivision throughout the whole brain and is therefore not system specific. In addition, anatomically defined subdivisions do not respect functional boundaries and vice versa, and each of the anatomically-defined amygdala subdivisions maintains connections along multiple pathways that are associated with various cognitive functions. In the present study, we therefore aimed to assess the functional architecture of the amygdala in adolescents with ASD by parcellating the amygdala based on its FC with three cortical seeds that are specifically anchored within the system of ‘social brain networks’ (SBNs).

Based on resting-state MRI scan data of healthy adults, Bickart et al. (2010, 2012) characterized three major SBNs that involved the amygdala: a ‘social perception’ network, a ‘social affiliation’ network and a ‘social avoidance’ network. For the ‘social perception’ network, the amygdala exhibited the strongest FC with the lateral orbitofrontal cortex (lOFC). The primary focus of amygdala connectivity for the ‘social affiliation’ network was found in ventromedial prefrontal cortex (vmPFC). Caudal anterior cingulate cortex (cACC) showed the strongest connectivity with the amygdala within the ‘social avoidance’ network. Bickart et al. (2010, 2012) used these cortical seed regions to parcellate the amygdala into three functional parcels: the ventrolateral, medial, and dorsal amygdala, respectively. Furthermore, the FC strength of these central nodes within the ‘social perception’ and ‘social affiliation’ networks correlated positively with the diversity and number of friends (Bickart et al. 2010, 2012).

Here, we first identified the three cortical seed regions found by Bickart et al. in our own adolescent healthy controls, and used these to parcellate the amygdala in both healthy controls and adolescents with ASD. We then compared the size of their associated parcels between the ASD group to the healthy controls. In posthoc analysis, we investigated these between-group differences further by delineating the relationship between the functional volumes and their FC strength to the three associated cortical seeds. Finally, we tested whether functional volume serves as a marker for social skills in ASD. Because the FC strength of the amygdala’s ‘vmPFC’ and ‘lOFC’ parcels have been reported as a good predictor of social network size (Bickart et al. 2010, 2012), we hypothesized that the volumes of the amygdala parcels predict the severity of social symptoms and impairment in ASD.

## Methods and Materials

### Participants

Twenty-one adolescents with autistic disorder according to the DSM-IV criteria and 25 typically developing controls were enrolled in the study. Participants with ASD were recruited through Karakter, Child and Adolescent Psychiatry University Center, Nijmegen. The study (including the informed consent procedure and all information brochures) was approved by both the regional ethics committee (Commissie Mensgebonden Onderzoek Arnhem Nijmegen) and Karakter’s review board. All participants provided verbal and written informed consent.

We only included participants with an intelligence quotient (full-scale IQ) of 80 or higher. Sixteen participants with ASD and 19 control participants under age of 18 completed the Wechsler Intelligence Scale for Children III (WISC-III) (Kort et al. 2002), while participants above age of 18 (ASD = 5, controls = 6) completed the Wechsler Adult Intelligence Scale III (WAIS-III) (Wechsler 2000). All participants also completed the short version of Edinburgh Handedness Inventory (Oldfield 1971).

All participants and their parents completed the Autism Spectrum Quotient (AQ) about themselves or their child respectively. The AQ is a validated measure of autism spectrum characteristics found within both the typical population and individuals with a diagnosis of ASD and thus provides a reliable measurement tool for the comparison of autistic traits between our ASD and control sample (Baron-Cohen et al. 2001).

Diagnoses of autistic disorder were based on a series of clinical assessments including a detailed developmental history, clinical observation, medical work-up and cognitive testing in a multidisciplinary team including a child psychiatrist and clinical psychologist. Diagnoses of autistic disorder was acquired with the Autism Diagnostic Interview-Revised (ADI-R) (Lord et al. 1994). We excluded those with ASD who had co-morbid psychiatric or neurological conditions including but not limited to attention deficit/hyperactivity disorder (ADHD), depressive disorder, schizophrenia, epilepsy or history of traumatic brain injury. None of the participants used medication.

Controls were matched at the group level on age, sex, and handedness and verbal, performance and full-scale IQ scores (Table 1). We ruled out the presence of psychiatric co-morbidity in controls and verified that all participants scored within the normal range using the school-age version of Child Behavior Check List (CBCL/6–18) and Adult Behavior Check List (ABCL/18–59).

**Table 1:** Subject demographics

	ASD	Control
Males	N = 19 (95%)	N = 22 (88%)
Females	N = 1 (5%)	N = 3 (12%)

	Mean	SD	Mean	SD	*p* value
Total IQ	102.30	13.57	103.72	9.78	0.69
Verbal IQ	101.00	13.37	104.60	11.29	0.35
Performal IQ	105.88	15.81	103.00	15.39	0.56
Age	16.23	3.18	16.11	2.79	0.90
Autism questionnaire (AQ)					
Participants	21.83	6.13	11.88	3.91	< 0.001*
Parents about participant	30.34	7.57	11.74	5.69	< 0.001*
Autism diagnostic interview (ADI-R)					
ADI-R A (10)	18.25	6.50			
ADI-R B (8)	15.70	5.54			
ADI-R C (3)	4.05	2.31			
ADI-R D (1)	2.65	1.35			

*p* value = *p* values indicate results for the independent t-test statistic. ADI-R (A) social interaction, (B) communication and language, (C) restricted and repetitive behavior (D) age of onset criterium; ADI-R thresholds are shown in parentheses. Pearson chi-squared for group by gender was non-significant (value = 0.672, df = 1, 2-sided asymptotic *p* = .412)

*Statistically significant

### Image Data Acquisition

For each participant, we acquired MRI data at the Donders Institute for Brain, Cognition and Behaviour, Center for Cognitive Neuroimaging in Nijmegen, The Netherlands, using a 3 T Magnetom TIM Trio (Siemens, Erlangen, Germany) with a 32-channel head coil. The entire scanning session lasted approximately 45 min. For each participant, we collected a T1-weighted whole-brain scan (magnetization-prepared rapid acquisition with gradient echo [MPRAGE], TI = 1100 ms, TR = 2300 ms, TE = 3.03 ms, flip angle = 8°, FOV = 256 × 256 × 192 mm^3^, voxel size = 1 × 1 × 1 mm^3^) and a resting-state scan using T2*-weighted dual-echo planar imaging (EPI, TR = 2510 ms, TE1 = 16 ms, TE2 = 36 ms, flip angle = 83°, FOV = 212 × 212 × 119 mm^3^, voxel size = 2 × 2 × 2.5 mm^3^, number of volumes = 400, imaging bandwidth = 1814 Hz/px, grappa acceleration factor = 4). Note that the usage of dual-echo imaging provides optimal sensitivity for BOLD imaging in both subcortical structures such as the amygdala and the neocortex (Poser et al. 2006). Participants were instructed to lie still within the scanner with their eyes open during the resting-state scan, while staying awake and focusing on a small white cross presented at the center of a projection screen. The first five volumes (12.55 s) were discarded to reduce magnetization equilibration effects. Gradient echo field mapping data were also acquired with identical geometry to the EPI data for EPI off-resonance distortion correction (TR = 1020 ms, TE1 = 10 ms, TE2 = 12.46 ms, flip angle = 90°, FOV = 224 × 224 × 191 mm^3^, voxel size = 3.5 × 3.5 × 2 mm^3^). All participants were able to familiarize themselves with scanner set-up and scanning procedure through rehearsal in a replicate (dummy) scanner before actual image acquisition.

We recorded participants’ heartbeats using the scanner’s built-in photoplethysmograph, placed on the right index finger. Respiration was measured with a pneumatic belt positioned at the level of the abdomen. To reduce the potential effects that heartbeat and respiration have on resting-state BOLD correlation studies (Birn et al. 2008; Chang et al. 2013), we used cardiac and respiratory phase regressors, as well as other nuisance regressors in the fMRI time series analysis.

### Preprocessing

All image preprocessing and analyses were performed using FSL (FMRIB Software Library, http://fmrib.ox.ac.uk/fsl) (Smith et al. 2004). The following pre-statistical processes were applied to the fMRI data: non-brain removal using BET; rigid-body motion correction using MCFLIRT; high-pass temporal filtering (Gaussian-weighted least-squares fitting with frequency cutoff point = 100 s); correction of off-resonance geometric distortions in the EPI data using PRELUDE and FUGUE, using B_0_ field maps derived from the dual-echo gradient echo dataset; artifact removal based on probabilistic ICA (Independent Component Analysis) using MELODIC; spatial normalization to Montreal Neurological Institute (MNI152) 2 mm isotropic atlas space using Boundary-Based-Registration (BBR) and FNIRT and Gaussian filtering (FWHM = 6 mm; see “Methods”). The dual-echo images (TE = 16 and TE = 36) were combined by averaging both echo-times. We excluded 1 participant with ASD due to excessive head movement in terms of frame-wise displacement (Max. FD = 8.7 mm, M_fd_ = 0.89 mm), resulting in 20 datasets from the ASD group and 25 datasets from the control group for further analysis (Rausch et al. 2016). To rule out that differences in movement between the ASD and control group could contribute to the results, we calculated the mean value of frame-wise movement (i.e. the movement of one TR relative to previous TR) for each participant and compared it between the two groups. No group difference was found (M_asd_ = 0.10, SD_asd_ = 0.10; M_ctrl_ = 0.07, SD_ctrl_ = 0.42; *t*_43_ = 0.312, *p* = .757).

### Controlling for Structured Noise

Our preprocessing stream included several steps to limit the influence of structured noise, such as motion artifacts (Power et al. 2012), heartbeat (Chang et al. 2013), and respiration (Birn et al. 2008). First, we conducted manual ICA-based artifact removal (Rausch et al. 2016). The first author visually inspected all the independent component maps for each participant to identify noise components based on the spatial layout of the component maps and the power spectra of the associated time-series (Kelly Jr et al. 2010). We applied non-aggressive denoising with FSL’s fsl_regfilt, i.e. only variance that was uniquely related to the components labeled as noise component (approx. 70% of components) was removed.

After ICA-based noise removal and further preprocessing, we conducted nuisance regression modeling the potential effect from motion and physiological noise on the resting-state fMRI data. Specifically, we included six rigid-body parameters and the eigenvariate of signals over the entire white matter and the CSF in our GLM. Moreover, we calculated ten cardiac phase regressors, ten respiratory phase regressors and six other nuisance regressors including heart rate fluctuation, heart rate variability, respiration raw data averaged per TR, respiratory amplitude in 9 s window, respiratory frequency in 9 s window and the frequency times amplitude of respiration (averaged per TR) that are derived from the RETROICOR method (Glover et al. 2000).

### Defining Cortical Seed Points

**Fig. 1:**
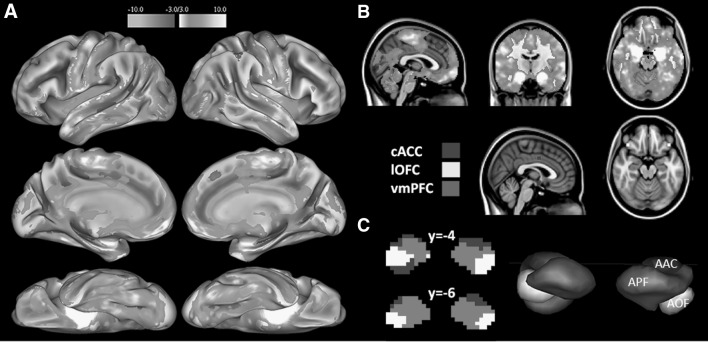
Spatial distribution of the three amygdalo-cortical networks from which cortical seed coordinates were defined and amygdala parcellation in controls. Panel **a** shows the t statistics for the amygdalo-cortical activation map from lateral, medial and ventral view on an inflated brain. Panel **b** shows the same from the coronal, saggital and axial view on a T1 MNI152 2 mm template brain in radiologic convention in the upper section. The lower section shows the cortical seeds positioned in the cACC, lOFC and vmPFC (cACC = ± 2, − 2, 38; lOFC = ± 40, 28, − 18; vmPFC = ± 2, 46, − 18). Cortical seeds were defined based on the full correlation maps with the entire amygdala in the control group. Panel **c** shows a visualization of the FC parcellation of the amygdala in the control group. Each voxel was assigned to the network with the maximum *Z*-value at the group-level. The left section shows the parcellation in 2d slices from two axes and the right section shows the parcellation using 3d rendering

We first extracted the mean time series from a bilateral amygdala mask (using FWHM = 1 mm Gaussian filtered functional images for the amygdala) and calculated its correlations with every voxel in the rest of the brain (using FWHM = 6 mm Gaussian filtered functional images for the whole brain) (Fig. 1a). Different smoothing kernels for the amygdala were used, because the width of the amygdala filter should be tailored to the parcel size between-group differences we expect to see (Rosenfeld 1976). The bilateral amygdala ROI was defined using probabilistic maps from the Harvard–Oxford Subcortical Structural Atlas available for FSL, limited to voxels that had 25% or greater probability of being labeled as the amygdala (left: 3392 mm^3^, right: 3888 mm^3^). We then identified peak voxels from the 1 − p statistical correlation map (*p* = .05) between the amygdala and areas within the boundaries of probabilistic vmPFC, lOFC and cACC maps within each hemisphere (Harvard–Oxford Structural Atlas) and created a 3 mm cortical sphere around the resulting six coordinates (cACC = ± 2, − 2, 38; lOFC = ± 40, 28, − 18; vmPFC = ± 2, 46, − 18). We then combined the coordinates to create three bilateral seed regions (lOFC, vmPFC and cACC), known to be involved in adaptive social behavior (Fig. 1b). The definition of the cortical seed regions was based solely on the control group.

### Parcellation of the Amygdala

We then parcellated the amygdala based on its FC with the three cortical seed regions. FSL’s SBCA was used to calculate the correlation between the mean time series of the voxels in each cortical seed and the time series of every voxel within the bilateral amygdala, corrected for the mean time series within the other two cortical seed regions. Thus, one single-subject partial correlation map of the amygdala for each hub within its network was obtained, which represented their unique connectivity with the amygdala. The partial correlation maps were *r*-to-*Z* transformed and each voxel was assigned to the network with the maximum *Z*-value. As a result, the amygdala was parcellated into three functional parcels: one parcel defined by maximal FC with the lOFC, one defined by maximal FC with the vmPFC, and one defined by maximal FC with cACC, which we will refer to as ‘AOF’, ‘APF’, and ‘AAC’ parcels respectively (Fig. 1c).

### Functional Amygdala Network Volume Analysis

To test whether the volume of the amygdaloid parcels differed between diagnostic groups, we extracted the three parcel volumes per subject and conducted second-level analysis in SPSS using ANCOVA (separate dependent variables: AOF, APF and AAC volume; fixed factors: diagnostic group; covariates: age and grey matter volume) (IBM Corp 2016). One participant was identified as an outlier using the standard definition of outliers as implemented in SPSS due to extreme values of the AAC parcel volume [N_ASD_ = 19; N_Ctr_ = 25; i.e., values outside of 1.5 times the interquartile range as implemented in SPSS 23 (IBM Corp 2016)] and excluded from the AAC parcel volume analysis, though removing this outlier did not change the statistical significance of the results. The data was normally distributed within the control and the ASD group [Shapiro–Wilk statistics: ASD_AAC_ (df = 19, *p* = .081); ASD_AOF_ (df = 20, *p* = .412); ASD_APF_ (df = 20, *p* = .222); CTR_AAC_ (df = 25, *p* = .950); CTR_AOF_ (df = 25, *p* = .610); CTR_APF_ (df = 25, *p* = .278)].

### FC Strength Posthoc Analysis

After assessing the FC volume of each parcel in ASD and controls, we examined the FC strength between the AOF, APF and AAC parcels with their corresponding cortical seeds in controls and ASD, which we will refer to as ‘FC_AOF–lOFC_’, ‘FC_APF–vmPFC_’ and ‘FC_AAC–cACC_’ strength respectively. To assess the direction of FC strength differences between both diagnostic groups, we extracted for each parcel the voxel-wise (Fisher *r*-to-Z transformed) partial correlations with their associated cortical target, and averaged these across all voxels in that parcel using SBCA [seed based correlation analysis (O’reilly et al. 2009)]. As we want to investigate those voxels that drove the differences between the ASD and control group, and because we do not expect to find any differences in FC strength in regions that belong to the same parcel in both groups, we used the average AOF, APF, and AAC parcels as defined within the control group as ROIs in this analysis. Note that the better parcel definition in controls as compared to the ASD group by itself might cause a bias towards higher FC strength in the control group. However, as the influence of neighboring parcels is regressed out using partial correlation analysis, and because our analysis approach therefore corrects for mixed signals between one parcel and its neighboring parcels in the ASD group, we thus corrected for the poorer parcel definition in the ASD group. Between-group comparison was carried out in SPSS using ANCOVA (separate dependent variables: The FC_AOF–lOFC_, FC_APF–vmPFC_ and FC_AAC–cACC_ strength; fixed factors: diagnostic group; covariates: age). Four participants were excluded in the FC_AOF–lOFC_ strength analysis and one participant was excluded from the FC_APF–vmPFC_ strength due to extreme values (FC_AOF–lOFC_ strength: N_ASD_ = 19; N_Ctr_ = 22; FC_APF–vmPFC_ strength: N_ASD_ = 20; N_Ctr_ = 24; i.e., according to the 1.5×IQR rule), though this did not change the statistical significance of the results. The data was normally distributed within the control and the ASD group [Shapiro–Wilk statistics: ASD FC_AAC–cACC_ (df = 20, *p* = .143); ASD FC_AOF–lOFC_ (df = 19, *p* = .808); ASD FC_APF–vmPFC_ (df = 20, *p* = .992); CTR FC_AAC–cACC_ (df = 25, *p* = .752); CTR FC_AOF–lOFC_ (df = 22, *p* = .879); CTR FC_APF–vmPFC_ (df = 24, *p* = .538)].

## Results

### Connectivity-Based Parcellation of the Amygdala in ASD Versus Controls

We first aimed to assess the functional architecture of the central hubs within the amygdala SBNs in ASD and controls. The amygdala was parcellated into three functional parcels (AOF, APF and AAC) based on its FC with each of the three cortical seeds (lOFC, vmPFC and cACC). Figure 1c shows the group-level parcellation for the controls, which corresponds well with the previous functional parcellation in healthy adults described by Bickart et al. (2012). The AAC, AOF and APF parcels yielded a mean (SD) volume across controls of 2031 mm^3^ (879.3 mm^3^), 2108.8 mm^3^ (883.4 mm^3^) and 3140.2 mm^3^ (1100 mm^3^) respectively. In the ASD group the mean parcel volume was 1576 mm^3^ (1244.2 mm^3^) in AAC, 3059.6 mm^3^ (1338.7 mm^3^) in AOF and 2454.8 mm^3^ (1231 mm^3^) in APF. To verify that this functional parcellation is based on meaningful signals, we confirmed that all parcels in both groups yielded significant FC with the corresponding cortical seeds (all *p* < .001 for both groups). Note however that the significance of these functional connections is to be expected given that we defined the cortical seeds by selecting those cortical locations as seed areas based on significant connectivity with the entire amygdala.

**Fig. 2:**
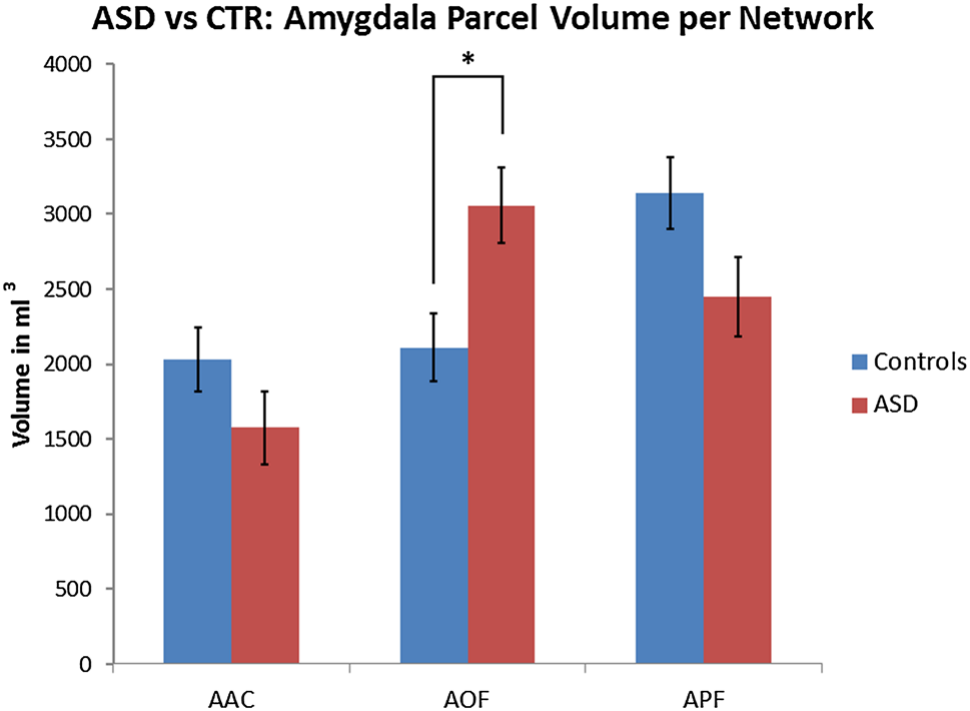
Amygdala parcel volume estimated marginal means in ASD and controls. The AOF parcel (middle) was significantly (*p* = .008) increased in ASD (red) compared to controls (blue), while there was a non-significant trend of decreased APF (right) and AAC (left) parcels in ASD compared to controls. Error-bars represent the standard error. (Color figure online)

We then compared the volume of the parcels between the ASD group and controls for each parcel individually. We found a significant increase in AOF parcel volume in the ASD group (F_1_ = 7.842, *p* = .008; age and total grey matter volume corrected) and a trend toward decreased APF volume (F_1_ = 3.794, *p* = .058) and AAC volume (F_1_ = 1.990, *p* = .166) (Fig. 2).

### Relationship Between Parcel Volume and Symptom Severity

To investigate whether the larger AOF parcel in ASD is associated with ASD social symptoms and its severity, we conducted correlation analysis (corrected for age and total grey matter volume) between the volume of AOF parcel and the social skills subdomain of the AQ. We found that AOF volume predicted symptom severity in the social skills subdomain (*r* = .548, df = 16, *p* = .009) of the ASD group.

### FC Strengths Posthoc Analysis Within Networks

**Fig. 3:**
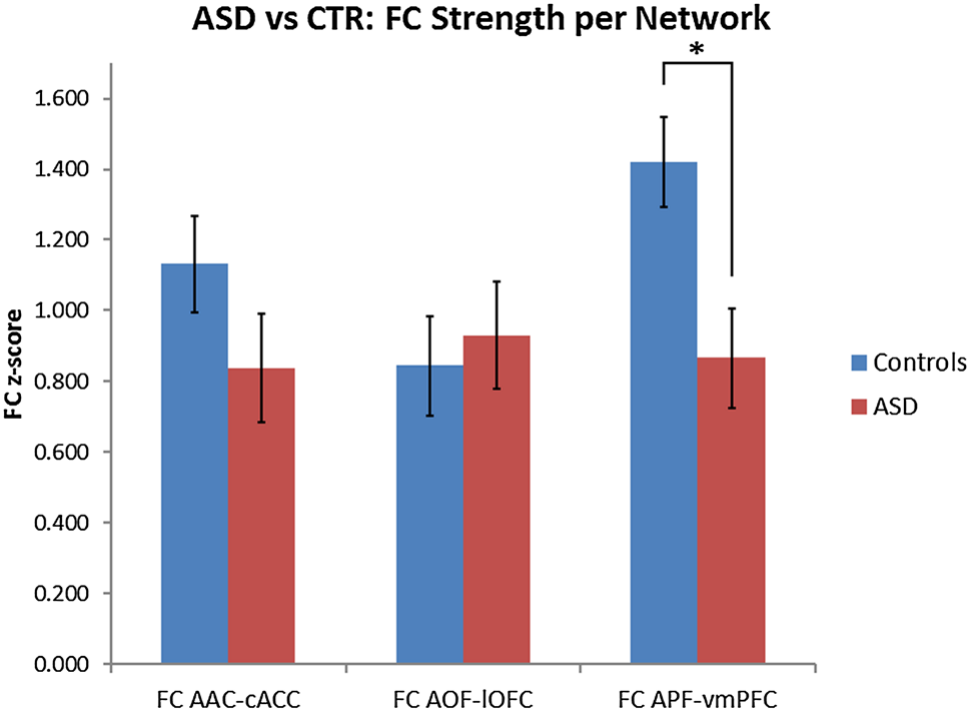
FC strength per parcel in the ASD group and controls. The FC_APF–vmPFC_ (right) FC strength was significantly decreased in ASD (red) compared to controls (blue), with a non-significant decrease in FC_AAC–cACC_ (left) and increase in FC_AOF–lOFC_ (middle) FC strength in ASD compared to controls. Error-bars represent the standard error. (Color figure online)

To understand the underlying biological mechanism that drives the volume changes in the ASD group, we assessed their FC strength (‘FC_AAC–cACC_’, ‘FC_AOF–lOFC_’ and ‘FC_APF–vmPFC_’) in a posthoc analysis. Our functional volume parcellation approach is based on a winner-takes-all method, which assigned one amygdala voxel to one central hub within one SBN. Thus, with this method, we reveal a FC map that is based on the strongest connectivity value with one SBN as compared to the other network hubs. By itself, this map does not contain information about the actual strength of connection with the winning SBN as it is based on the relative strength between networks within a diagnostic group. The FC strength measure, on the other hand, enables us to investigate whether the AOF volume group difference was driven by abnormalities (i.e. FC strength increases) originating directly within its corresponding social perception SBN, or whether the AOF volume group difference was driven by alterations (i.e. FC strength decreases) within one or both of the neighboring SBN’s. Therefore, we assessed whether the increase in volume of the AOF parcel could be the result of two different mechanisms: (1) increased FC_AOF–lOFC_ strength in the ASD group, or (2) decreased FC strengths in one or two of the other networks hubs in the ASD group. To disambiguate which of these two is driving the AOF volume difference, we tested the direction of FC strength change for each SBN. The results show a decrease in FC_APF–vmPFC_ strength (F_1_ = 8.596, *p* = .005), but no between-group effects in FC_AAC–cACC_ strength (F_1_ = 2.022, *p* = .162) and FC_AOF–lOFC_ strength (F_1_ = 0.170, *p* = .682) (Fig. 3). These results indicate that the increase in volume of the AOF parcel originated from the APF parcel, because the FC strength between AOF and lOFC is constant between ASD and controls, while the FC strength between APF and vmPFC was lower for the ASD group compared with controls (Fig. 3).

**Fig. 4:**
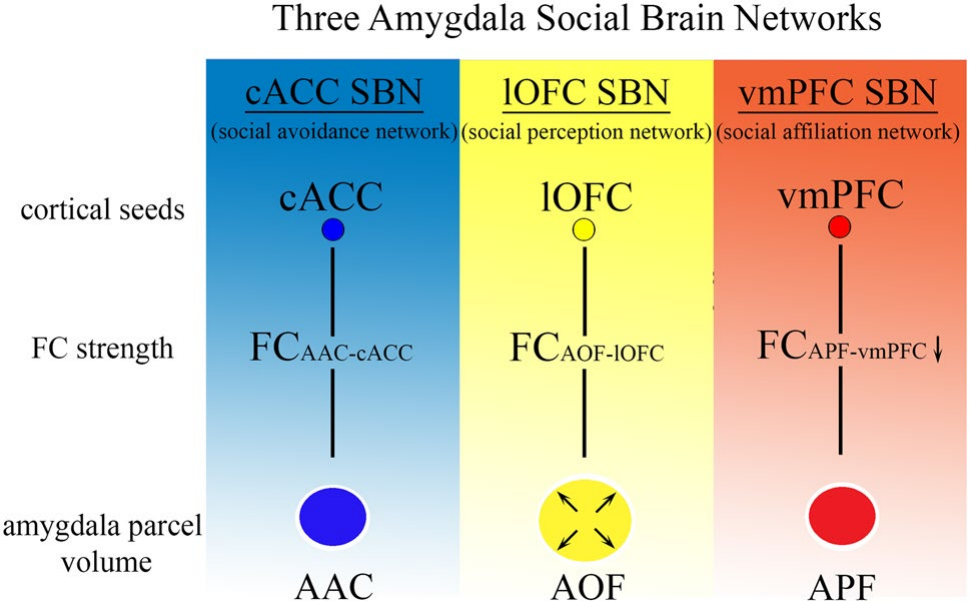
Schematic overview of the three amygdala SBNs. The blue, yellow and red panels show the cortical seeds, FC strength parameters and amygdala parcel volume parameters per amygdala social brain network (SBN). Arrows indicate increases or decreases in FC within an SBN. In the ASD group, AOF parcel volume was increased in ASD, while FC_APF–vmPFC_ strength was reduced. (Color figure online)

To investigate whether the reduced FC_APF–vmPFC_ strength in ASD is associated with ASD social symptoms and its severity, we conducted a correlation analysis (corrected for age) between the FC_APF–vmPFC_ strength and the social skills subdomain of the AQ, which was not significant (*r* = − .087, df = 17, *p* = .362). Furthermore, though we only found a weak trend towards reduced FC_AAC–cACC_ strength, the AOF parcel enlargements may in part relate to the marginally significant AAC parcel reductions. Therefore, the combined FC_APF–vmPFC_ and FC_AAC–cACC_ strength might explain more variance of the social symptoms scores in ASD than the FC_APF–vmPFC_ strength alone. Thus, we assessed whether their combined FC strength could predict social AQ scores using linear regression analysis (corrected for age). The result was non-significant (F_3_ = 2.082, *p* = .143). Figure 4 contains a schematic overview of the main results.

## Discussion

We assessed the functional architecture of the amygdala in adolescents with ASD by parcellating the amygdala based on its FC with three cortical seeds (cACC, lOFC and vmPFC) that are anchored within known SBNs: the social avoidance network, the social perception network and the social affiliation network. Three functional parcels were created (AAC, AOF and APF) based on its FC with each of the three cortical seeds respectively. We found a significant enlargement of the AOF parcels in the ASD group, while there was a trend toward decreased volume of the other two parcels in ASD, especially of the APF parcel. We assessed the clinical relevance of our marker, and found that increased AOF parcel volume predicted impairments in social skills in the ASD group. In posthoc analysis, we found that the increase of the AOF parcel came at the cost of the APF parcel, as indicated by a decrease in FC_APF–vmPFC_ strength.

Our results align well with and extend earlier findings showing that especially the lOFC and vmPFC amygdala SBNs predicted social outcome in a healthy control sample (Bickart et al. 2012). All three affective networks are associated with generating appropriate adaptive social behavior and are known to work together closely (Bickart et al. 2014). We show for the first time that ASD is associated with FC abnormalities of the lOFC and vmPFC amygdala pathways, which are the central hubs of social perception and social affiliation network functionality. The AOF parcel roughly corresponds to the ventrolateral subregion containing the laterobasal nucleus of the amygdala and the APF parcel to the medial subregion containing the superficial nucleus according to the probabilistic cytoarchitectonically defined Jülich atlas (Amunts et al. 2005), which are associated with perceptual input processing of the amygdala (Rausch et al. 2016). Although the AAC parcel roughly corresponds to the dorsal amygdala containing the centromedial amygdala nucleus, Fig. 1c indicates hemispheric lateralization related to the AAC parcel. More specifically, the right AAC extends into the ventrolateral subregion, which has been assigned to the AOF parcel in the left hemisphere. The literature on lateralization effects on subregion level in the amygdala is however sparse, and it is therefore unclear what the functional implications of this lateralization effect are (Gläscher and Adolphs 2003; Gorka et al. 2017; McMenamin and Marsolek 2013). The AAC did not show any group effects in the FC strength analysis or parcel volume in our study. Our findings therefore may be in line with an earlier study investigating abnormalities in three anatomically defined amygdala subdivisions (the laterobasal, superficial and centromedial nuclei) that implicated amygdala under-connectivity between the superficial and laterobasal nuclei and cortex (Rausch et al. 2016).

Given the inhibitory relationship between the vmPFC and the amygdala in the literature (Motzkin et al. 2015), our findings of reduced FC_APF–vmPFC_ strength may point toward weaker inhibitory connections of the vmPFC amygdaloid circuit in ASD. Our posthoc FC strength analysis also demonstrates that a potential lack of inhibition from the vmPFC onto the amygdala in ASD is not linked to significantly increased FC strength of the lOFC amygdaloid network, because the FC_APF–vmPFC_ strength decrease was not accompanied by a significant increase in FC_AOF–lOFC_ strength. However, our results might indicate that a potential lack of inhibition from the vmPFC might be driving a weak—but not significant—increase of FC_AOF–lOFC_ strength in ASD, that is spanning a significantly larger area in the ASD group as compared to controls. Recent findings suggest that abnormalities in FC strength are rather characterized by a diffuse distribution of FC in ASD (Hahamy et al. 2015). In other words, the autistic brain may have idiosyncratic FC patterns, which cannot be identified in terms of a “common spatial locus” of abnormal FC strength changes. Instead, the areas in which the FC abnormalities occur, might be characterized by different FC between ASD subjects, which might partially explain diffuse and widespread FC changes in ASD. This suggests that ASD is not a disorder of unique abnormal loci per se, but rather a problem of the functional specialization as compared to controls. Therefore, because our functional volume measures provide a quantification of FC that is independent of a “common spatial locus” of abnormal activation within the amygdala (yet tied to specific functionality), our functional volume measure might indicate that alterations of the social perception lOFC amygdala network, are characterized by abnormal FC distribution.

We also assessed how well our FC markers predict social skills based on the AQ social subdomain in the ASD group. Since the increase of the AOF volume may be a consequence of decreased FC_APF–vmPFC_ strength, the correlation between decreased FC_APF–vmPFC_ strength and social skills was tested, but was not significant. However, we were able to relate our findings of increased AOF volume to social skills. Because the increase in AOF volume appears to be a consequence of decreased FC_APF–vmPFC_ strength, and because the AOF volume significantly predicted social skills in the ASD group, it might be surprising that we did not find significant negative relationships between social skills and the FC strength of the APF (and/or ACC) parcel(s). The most parsimonious explanation for this apparent inconsistency is that parcel volume and FC strength measure different things and that the first better probes the underlying pathology than the latter. A parcel’s volume is dependent on the number of voxels that exhibited maximum partial correlation with that parcel’s cortical target. Maximum partial correlation can be achieved by very small correlation differences, so for instance a relatively large parcel volume can be due to having many voxels with very small correlation differences. As such, differences in parcel volume can be great while the difference in average FC strength is very small. In other words, parcel volume, though derived from FC strength estimates, does not have to follow the same pattern as the average FC strength. The fact that parcel volume better predicts social skills than average FC strength can further be taken to imply that the underlying pathology can be attributed to a large number of voxels (neurons) that exhibit an abnormal balance in terms of its connectivity with the three cortical targets with only subtle alterations in the strength of these connections. Therefore, our functional parcel volume approach may provide a sensitive alternative to standard thresholding techniques for capturing subtle functional changes in the architecture of FC in ASD.

Reduced amygdaloid vmPFC strength link our functional volume abnormalities to results showing under-connectivity patterns in ASD populations. As the vmPFC is part of the mentalizing or the theory of mind network, person perception, self-knowledge (Amodio and Frith. 2006) and the processing of pleasant outcomes like social and monetary rewards (Rademacher et al. 2010) our results align well with the idea that FC along the amygdala-vmPFC pathway might be altered in ASD. One study showed that the dorsal medial PFC is activated rather than the ventral medial PFC in ASD during a self-referential task, which suggests under-connectivity of the vmPFC in ASD (Schulte-Rüther et al. 2011). Another study found amygdalo-vmPFC under-connectivity when viewing sad faces in ASD (Swartz et al. 2013). Yet another study investigated oxytocin-induced activation, i.e. a crucial hormone in affective processing, in the vmPFC and pointed to an oxytocin-induced activation increase in the vmPFC and that this effect furthermore improved socio-communication difficulties in ASD (Aoki et al. 2015). Thus, our findings of decreased FC_APF–vmPFC_ strength are consistent with the known abnormalities along the amygdala-vmPFC pathway in ASD.

The amygdala is known to be a complex subcortical structure with many efferent and afferent subcortical and cortical as well as intra amygdala connections. Therefore, investigating amygdala functional connections using the entire amygdala does not account for its complex underlying pathways. One more fine grained method to investigate abnormal amygdala functional connections is to parcellate the amygdala based on anatomical subregions within the amygdala (Rausch et al. 2016; Ball et al. 2007; Roy et al. 2009) to assess subregion specific abnormalities. This method is however restricted to predefined anatomical ROI’s based on healthy adult brains, which furthermore are associated to multiple functional pathways. In the current study, therefore we parcellated the amygdala into three functionally defined network parcels based on known amygdala SBNs within our own adolescent sample. This way, we established functionally meaningful amygdaloid subregions in our control sample, which were used to characterize the FC within three important amygdala SBNs in controls and our ASD group.

One potential limitation of our study is the use of a small homogenous sample. Our ASD sample does not include individuals with highly prevalent co-morbidities in ASD such as anxiety, depression or ADHD. No individuals with PDD–NOS were included in our sample. Therefore, our results only provide evidence for autistic core features. Although there was no trend towards group effects in FC_AOF–lOFC_ (*p* = .682) or FC_AAC–cACC_ (*p* = .162) strength, future work involving larger ASD samples may be able to stratify the FC of the SBNs according to age, gender, and symptoms (Murphy and Spooren 2012). In addition, in order to maximize sensitivity of social measures for predicting functional volume, AQ measures may be complemented with interview data (Vineland) and observational measurements (ADOS), which could not be included in the present work as they were deemed too demanding for the ASD group who had already been diagnosed at the time of the study.

Our results demonstrate that functional amygdala parcellation based on its FC with three major amygdala SBNs is a sensitive measure for capturing the functional architecture of dysfunctional amygdalocortical pathways in ASD. Within the three SBNs that were investigated within this study, our results suggest that underconnectivity between amygdala and prefrontal vmPFC is driving abnormal functional interactions between the amygdala and other amygdala networks. By parcellating the amygdala functionally into volumes pathophysiological mechanisms along the amygdalo-prefrontal pathway could be linked to increasing symptom severity in ASD.

## Abbreviations

SBN: Social brain network
cACC: Caudal anterior cingulated cortex
lOFC: Lateral orbitofrontal cortex
vmPFC: Ventromedial prefrontal cortex
AOF parcel: Amygdala lOFC parcel
APF parcel: Amygdala vmPFC parcel
AAC parcel: Amygdala cACC parcel
FC_AOF–lOFC_: Functional connectivity between AOF and lOFC
FC_APF–vmPFC_: Functional connectivity between APF and vmPFC
FC_AAC–cACC_: Functional connectivity between AAC and cACC
